# The Role of Mothers’ Cognitive Health In Sibling Support, Strain and Psychological Well-Being During Caregiving

**DOI:** 10.1093/geroni/igaf122.2359

**Published:** 2025-12-31

**Authors:** J Jill Suitor, Megan Gilligan, Robert Frase, Ranran He, Destiny Ogle, Di Wang, Hanamori Skoblow, Viridian Verbanac

**Affiliations:** Purdue University, West Lafayette, Indiana, United States; University of Missouri, Columbia, Missouri, United States; Southern Illinois University, Carbondale, Illinois, United States; Purdue University, West Lafayette, Indiana, United States; Purdue University, West Lafayette, Indiana, United States; Purdue University, West Lafayette, Indiana, United States; University of Missouri, Columbia, Missouri, United States; Purdue University, West Lafayette, Indiana, United States

## Abstract

A growing number of studies have shown that caregiving is usually a “family affair” navigated among siblings. Most of this research has focused on the detrimental consequences of negative interactions with siblings. As a result, less is known about the potential benefits of sibling ties for caregivers. We extend previous research by considering the differential impact of sibling support and strain on psychological well-being in the context of maternal care provision. We use mixed-methods data collected from 307 adult children nested within 150 families collected as part of the Within-Family Differences Study-III. On average, these adult children were 59 years old, and their mothers were 89 years old. We considered whether the impact of sibling support (i.e. siblings create feelings of love and care) and strain (i.e. siblings create arguments and disagreements) on psychological well-being varied by whether older mothers had symptoms of cognitive impairment (CI). Findings indicated that when adult children provided care to mothers who did not have symptoms of CI, sibling support was associated with increased psychological well-being, whereas sibling strain had no effect. In contrast, when adult children provided care to mothers with symptoms of CI, sibling strain was associated with decreased psychological well-being, whereas sibling support had no impact. These findings suggest that when mothers do not have CI, sibling support is beneficial to caregivers’ psychological well-being; however, when CI is present, caregivers do not experience these benefits from sibling’s positive interactions, yet experience the detrimental effects of negative interactions.

